# Supplementary material for: Imaging antiferromagnetic antiphase domain boundaries using magnetic Bragg diffraction phase contrast
Source: Nat Commun. 2018 Nov 27;9:5013. doi: 10.1038/s41467-018-07350-3 (PMC6258669; doi:10.1038/s41467-018-07350-3)
Supplement: Supplementary file 1 — Supplementary Information [file 41467_2018_7350_MOESM1_ESM.pdf]

# Supplementary Information

**Imaging antiferromagnetic antiphase domain boundaries using magnetic Bragg diffraction phase contrast**

Kim at al.

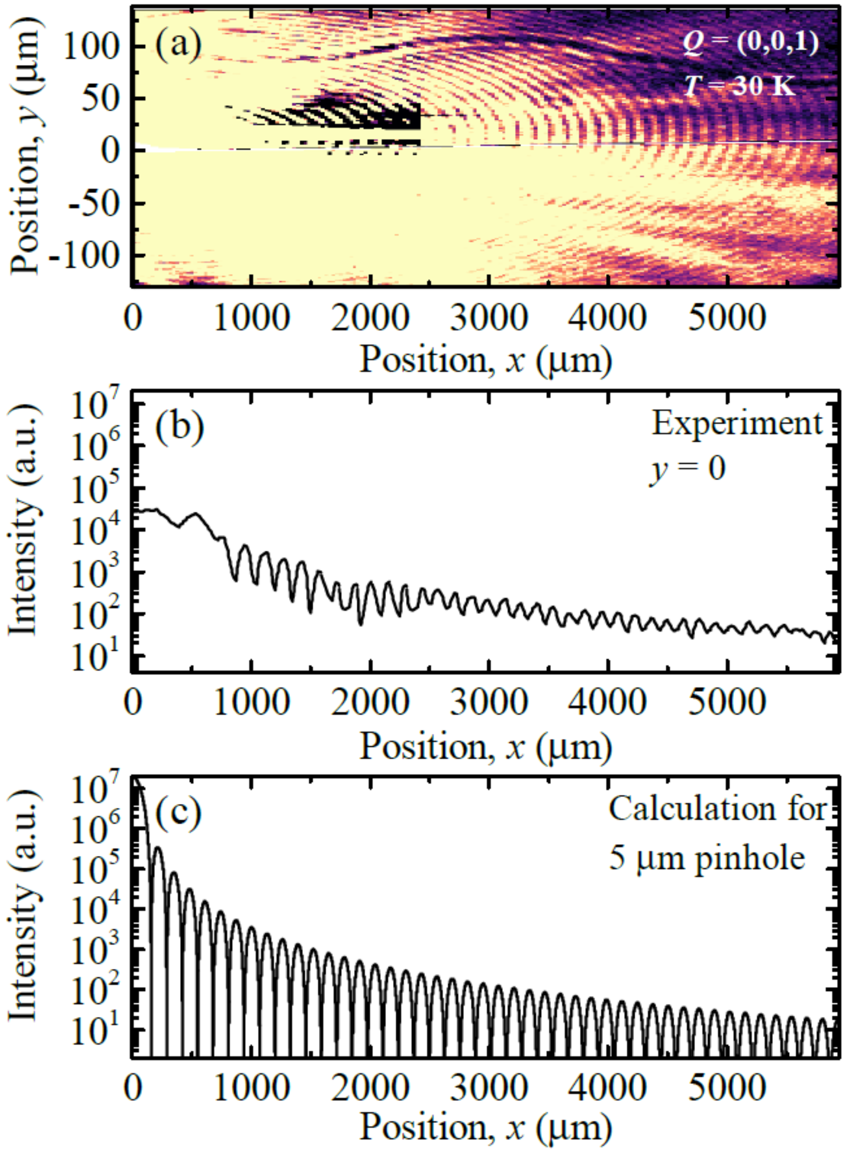


**Supplementary Fig. 1**. (a) A blowup of the diffraction fringe pattern produced by the pinhole, reflected off the sample surface in specular geometry, and observed on the detector. The central Airy spot is in the saturated central detector region, $x$=$y$=0. Numerous bright and dark rings are observed. In this figure, the axes refer to the position on the area detector. (b) Intensity line cut from the image in (a) taken at $y$=0. (c) Calculated intensity profile for an ideal 5 μm circular pinhole, showing a good match to the experimental data.

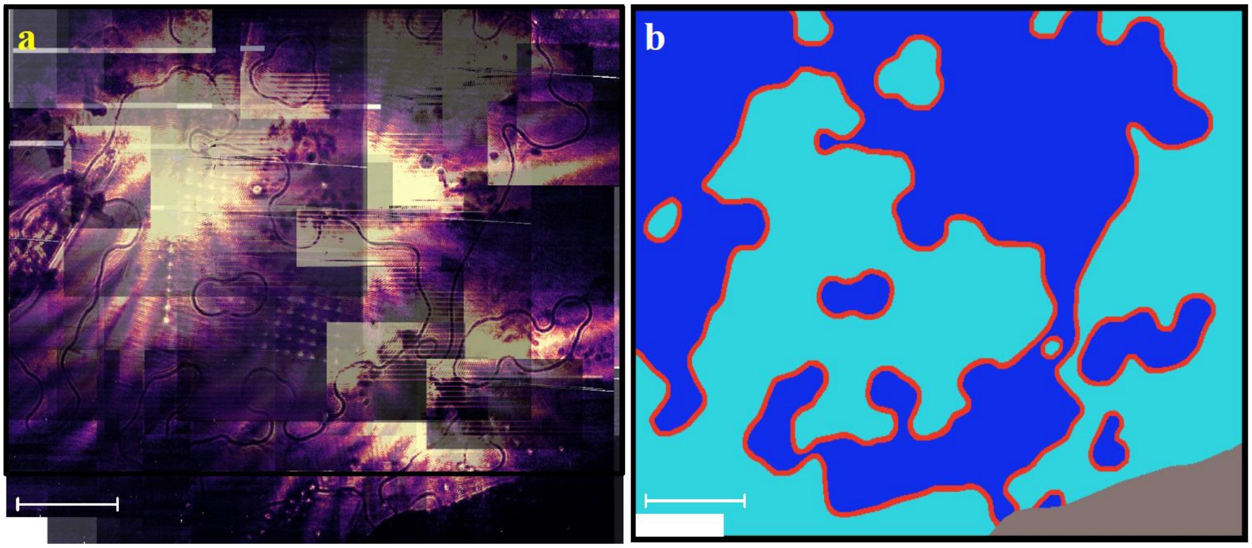


**Supplementary Fig. 2.** (a) Image of AFM antiphase domain boundaries, stitched together from numerous single-shot images taken for different incident beam positions on the sample surface. The images are taken under the same conditions as those shown in Figs 3(a), 4, and 5. Absence of the scattering signal in the lower right corner signifies the sample edge. The origin of regularly-spaced bright dots is discussed in the text. (b) Reconstructed magnetic domain pattern. The area beyond the sample edge is shown in grey. Scale bars (100 μm) refer to the distances on the sample surface.

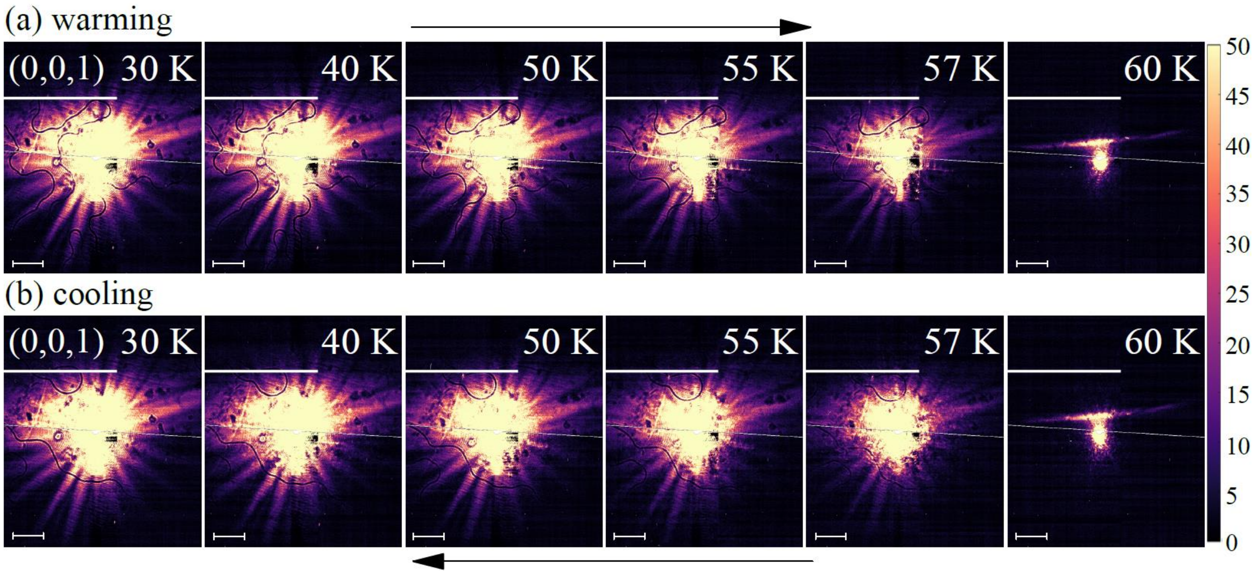


**Supplementary Fig. 3.** Single-shot images at the (0,0,1) magnetic Bragg peak position, taken on warming from 30 K to above the Néel temperature (a), and on subsequent cooling (b). Horizontal scale bars (50 μm) refer to the distances on the sample surface. The color scale-bar units are arbitrary.

## Supplementary Note 1

**Magnetic domain wall width estimate.** For a 180$^o$ domain wall in a simple uniaxial magnet, the Bloch wall width $W$ is given by $W = \pi S \sqrt{\frac{J}{K_1^u a}}$ where $S$ is the spin, $J$ is the magnetic exchange constant, $a$ is the distance between the spins, and $K_1^u$ is the magnetic anisotropy energy. In $Fe_2Mo_3O_8$, the average spin is 2.25, and the Curie-Weiss temperature $\theta$ is $-110$ K. In the mean field approximation, $\theta = \frac{1}{3}S(S+1)J$, giving the effective $J$≈45 K. $Fe_2Mo_3O_8$ is a very anisotropic uniaxial magnet. The local magnetic fields for the two Fe sites in this compound derived from Mössbauer measurements [1] are 58 and 183 kOe. This gives the average magnetic anisotropy energy $K_1^u$≈35 K. As a result, the estimate for the magnetic wall width is $W$≈3$a$≈1 nm. While this estimate is very crude, it is clear that the magnetic domain wall width in a highly anisotropic magnet $Fe_2Mo_3O_8$ should be negligibly small compared to the μm-scale experimental resolution.

## Supplementary References

1. Varret, F., Czeskleba, H., Hartmann-Boutron, F. & Imbert, P. Étude par effet Mössbauer de l'ion $Fe^{2+}$ en symétrie trigonale dans les composés du type (Fe, M)$_2$Mo$_3$O$_8$ (M = Mg, Zn, Mn, Co, Ni) et propriétés magnétiques de (Fe,Zn)$_2$Mo$_3$O$_8$. *J. Phys. France* **33**, 549-564 (1972).
